# Query-Efficient Hard-Label Attack: A Prior-Guided Adam Ray Search Optimization

**DOI:** 10.3390/s26103272

**Published:** 2026-05-21

**Authors:** Tianyi Ding, Xinjie Xu, Qi Xuan, Hanzhe Yu, Chen Ma

**Affiliations:** 1 Institute of Cyberspace Security, Zhejiang University of Technology, Hangzhou 310023, China; dingtianyi6@gmail.com (T.D.); xuanqi@zjut.edu.cn (Q.X.);; 2 Binjiang Institute of Artificial Intelligence, Zhejiang University of Technology, Hangzhou 310056, China

**Keywords:** black-box adversarial attack, Adam optimization, adversarial examples, hard-label attacks, AI security

## Abstract

Deep neural networks are vulnerable to adversarial examples, even in hard-label black-box settings where only the top-1 prediction is available. To address the challenges of high-dimensional optimization under limited query budgets, we propose two query-efficient attack methods: Adam-OPT, which integrates Adam-based adaptive optimization into the ray-search framework to stabilize and accelerate zeroth-order gradient updates; Prior-Adam-OPT, which further incorporates transfer-based priors from surrogate models to enhance gradient estimation. Adam-OPT leverages historical gradient information and per-parameter adaptive updates to improve convergence, while Prior-Adam-OPT constructs a prior-guided orthogonal search basis that combines surrogate and random directions, enhancing both gradient accuracy and query efficiency. Our approach demonstrates superior performance across CIFAR-10, ImageNet, and zero-shot CLIP models, consistently reducing perturbation magnitudes and improving attack efficiency compared to state-of-the-art hard-label attacks. Ablation studies highlight the importance of the number of vectors used for gradient estimation and the quality of surrogate models, showing that combining adaptive optimization with transfer-based priors provides a scalable and robust framework for generating high-quality adversarial examples in challenging black-box scenarios.

## 1. Introduction

Over the past decade, deep neural networks (DNNs) have made remarkable progress in areas such as object recognition, autonomous systems, and face recognition, achieving unprecedented classification accuracy. In particular, deep learning has demonstrated outstanding performance in visual tasks, including image classification, object detection, and image segmentation. However, as these technologies continue to evolve, DNNs face increasingly sophisticated security threats. Research has shown that DNNs are highly vulnerable to carefully crafted adversarial examples, which are generated by adding subtle and often imperceptible perturbations to original images. Despite their minimal magnitude, these perturbations can cause DNNs to produce incorrect predictions or classification results. Early studies on adversarial attacks [1] mainly focused on the white-box setting, where adversaries have full access to the target model’s architecture, parameters, and gradient information. Nevertheless, such assumptions are often impractical in real-world applications, motivating growing attention toward black-box attack scenarios, where internal model information, such as parameters and gradients, is inaccessible to the adversary. Among them, hard-label attacks are considered one of the most restrictive and challenging settings, since the adversary can access only the final top-1 predicted label without any confidence scores or probability distributions, which is different from typical score-based attacks [2]. This highly limited feedback setting constitutes the primary focus of this paper.

In hard-label attacks, predictions change only near the decision boundary, resulting in a highly discontinuous objective function. Consequently, the attack process involves solving a highly challenging, high-dimensional combinatorial optimization problem. Existing hard-label attack methods typically rely on random walk strategies or gradient estimation to search for adversarial directions. These approaches generally start from a sample with large adversarial perturbations and iteratively reduce distortion by moving along the decision boundary toward the original image. However, such methods often suffer from high query complexity and lack theoretical convergence guarantees. Excessive queries may also increase the risk of being detected by defense or monitoring systems, thereby leading to attack failure. This challenge becomes particularly severe in high-dimensional spaces, such as ImageNet, where the search space for adversarial perturbations is extremely large.

A representative class of hard-label attacks, namely the OPT-series methods [3,4,5,6], formulates hard-label attacks as continuous optimization problems. Representative approaches, including OPT [3], Sign-OPT [4], Prior-OPT [5], and PARS-OPT [6], optimize a search direction θ to minimize the $\ell_{p}$-norm distance from a benign image to the adversarial region. For example, OPT employs a randomized gradient-free (RGF) [7] estimator. However, each function evaluation required by finite-difference gradient estimation involves a costly binary search to locate the decision boundary, resulting in substantial query overhead. Sign-OPT [4] reduces query complexity by leveraging the sign of directional derivatives, but at the cost of degraded gradient accuracy. Prior-OPT [5] further improves efficiency by incorporating transfer-based priors into gradient estimation, thereby alleviating the poor estimation quality caused by purely random directions. However, it still relies on simple gradient descent updates and makes limited use of historical gradient information, leading to suboptimal convergence under tight query budgets. Overall, existing methods still largely rely on simple optimization strategies and fail to fully exploit advanced optimization algorithms, such as Adam [8], to accelerate convergence and improve query efficiency.

To address the aforementioned challenges, this paper proposes Adam-OPT and its prior-guided variant, Prior-Adam-OPT. The proposed method employs an Adam-based optimization strategy to improve the convergence and stability of ray-search optimization. By leveraging momentum and adaptive update mechanisms, Adam helps smooth noisy gradient estimates and guide the search direction more effectively. After each update, the direction is normalized to satisfy the unit-norm constraint, ensuring that the optimization is performed over the directional space rather than the perturbation magnitude. Building on this framework, Prior-Adam-OPT incorporates surrogate-model gradients as transfer-based priors. These priors are combined with random directions and orthogonalized to construct a more informative search basis, thereby improving the accuracy of gradient estimation and enhancing query efficiency under limited query budgets.

The main contributions of this paper are as follows:

- We introduce an Adam-based ray-search optimization strategy that decouples perturbation direction and magnitude. The search direction is updated using Adam with historical gradient information, normalized to satisfy the unit-norm constraint, and then evaluated by binary search to obtain the corresponding boundary distance. This adaptive update mechanism stabilizes noisy zeroth-order gradient estimation and improves convergence and query efficiency under limited query budgets.
- We further improve Adam-based direction optimization by incorporating surrogate gradient priors into the gradient estimation process. The prior-guided orthogonal basis provides more reliable update directions for Adam, enabling its momentum and adaptive update mechanisms to accumulate useful historical information more effectively. This design enables faster and more stable direction updates, thereby improving query efficiency under limited query budgets.
- Experimental results on standard benchmarks, including CIFAR-10 [9] and ImageNet [10], show that Prior-Adam-OPT achieves better query efficiency and lower perturbation distortion than existing hard-label attacks, such as Sign-OPT and Prior-OPT.

## 2. Related Work

Research on geometric optimization and evolutionary strategies has yielded significant advancements. The authors of Dong et al. [11] proposed the evolutionary algorithm, which employs a black-box optimization method based on the Covariance Matrix Adaptation Evolution Strategy (CMA-ES) to find optimal solutions by adding random noise and performing local searches. The authors of Rahmati et al. [12] proposed GeoDA, which leverages the property in which the average curvature of the deep network decision boundary is small near samples. By estimating normals with few queries, it designs a query-efficient $\ell_{p}$-norm attack. The authors of Maho et al. [13] proposed SurFree, a black-box adversarial attack algorithm based on geometric principles. By constructing a hyperplane and searching for closer points within it, it avoids expensive gradient proxy estimation. The authors of Reza et al. [14] proposed the curvature-aware geometric decision-based black-box attack algorithm CGBA and its variant CGBA-H. This algorithm searches along a semicircular path on a constrained 2D plane, ensuring that boundary points are found without being affected by boundary curvature. The authors of Ma et al. [15] proposed Tangent Attack (TA), a geometry-inspired hard-label attack that constructs a virtual hemisphere around the current adversarial point and searches along tangent directions on this hemisphere to efficiently reduce $\ell_{2}$ distortion.

In the realm of optimization and ray search, notable contributions have also been made. The HopSkipJumpAttack (HSJA) [16] initializes with target class samples, pushes them towards the boundary via binary search, and estimates the gradient direction, gradually reducing the step size to optimize $\ell_{2}$ and $\ell_{\infty }$ distances, significantly improving query efficiency. The ray search attack RayS [17] proposed by Chen and Gu relies solely on hard-label outputs. It performs a binary search on discrete ray directions to find the local optimum of the decision boundary radius, greatly reducing the number of queries. Its hierarchical search version exploits spatial correlations to search on small patches, thereby improving efficiency, and has become one of the baselines for current hard-label attacks.

Additionally, reinforcement learning and adaptive strategies have been integrated into attack methodologies. The authors of Yan et al. [18] proposed PDA (Policy Driven Attack), a decision-based black-box attack using reinforcement learning. It samples direction vectors from a normal distribution and performs jump searches based on rewards to select the optimal direction. The authors of Li et al. [19] proposed a hard-label black-box attack method, the adaptive history-driven attack, which dynamically adjusts based on historical query information. By adaptively optimizing direction coefficients to balance the weights between source and spherical directions, it effectively reduces perturbation magnitudes. The authors of Shi et al. [20] proposed patch-wise adversarial removal (PAR), a decision-based black-box attack for Vision Transformers that reduces query complexity by iteratively compressing perturbations on less sensitive patches.

## 3. The Proposed Approach

### 3.1. Problem Definition of Hard Label Attacks

In the hard-label black-box setting, the adversary has access only to the top-1 predicted label of the target classifier, and the objective is to find a minimal perturbation that induces misclassification. Given a classifier $\psi :R^{d}\to R^{C}$ for a *C*-class classification task, and a benign input sample $x\in {\left[0,1\right]}^{d}$ with ground-truth label *y*, the attack can be formulated as the following constrained optimization problem:

(1) $$\min_{x_{\mathrm{adv}}}\;{∥x_{\mathrm{adv}}-x∥}_{p}\;s.t.\;\;\Phi \left(x_{\mathrm{adv}}\right)=1,\;$$

where Φ(·) is the adversarial success indicator function defined as

(2) $$\Phi \left(x_{\mathrm{adv}}\right):=\left\{\begin{array}{cc} 1 & \mathrm{if}\;\hat{y}=y_{\mathrm{adv}}\;\mathrm{in}\;a\;\mathrm{targeted}\;\mathrm{attack},\\ \; & \;\mathrm{or}\;\hat{y}\neq y\;\mathrm{in}\;\mathrm{an}\;\mathrm{untargeted}\;\mathrm{attack},\\ 0 & \mathrm{otherwise}. \end{array}\right.\;$$

where $\hat{y}$ is obtained as the class with the largest output logits of ψ on the adversarial example, i.e., $\hat{y}=\arg\max_{i\in 1,\ldots ,C}\psi {\left(x_{\mathrm{adv}}\right)}_{i}$. Here, *y* denotes the ground-truth label of the benign input x, while $y_{\mathrm{adv}}$ represents the desired target class in the targeted attack setting.

From an optimization perspective, the objective defined in Equation (1) seeks the closest adversarial example to the original input. However, in the hard-label black-box setting, neither gradient information nor confidence scores are available, making direct optimization intractable. To address this challenge, we adopt the ray-search formulation introduced by OPT-series methods [3,4,5,6], which optimizes the search direction θ to minimize the boundary distance f(θ) to the adversarial region. This can be formulated as:

(3) $$\begin{array}{cc} \min_{\theta \in R^{d}\setminus \left\{0\right\}}\; & f(\theta ),\\ \mathrm{where}\;\; & f\left(\theta \right):=\inf\left\{\lambda >0:\Phi \left(x+\lambda \frac{\theta }{∥\theta ∥}\right)=1\right\}. \end{array}\;$$

When no adversarial point can be found along direction θ, the function value is set to f(θ)=+∞. Let θ* denote the optimal direction obtained by solving Equation (3); the corresponding adversarial example is then given by $x*=x+f\left(\theta *\right)\frac{\theta *}{∥\theta *∥}$, where θ* is the optimal solution obtained from the minimization problem defined in Equation (3).

### 3.2. Adam Optimization for Ray Direction Search

#### 3.2.1. Overview

This section introduces Adam-OPT, a hard-label black-box attack method that improves the ray-search paradigm with Adam-based adaptive optimization. Under the ray-search formulation, the perturbation is decomposed into a unit search direction and a boundary distance. Adam-OPT optimizes the search direction, while the corresponding boundary distance is determined by binary search along the current ray direction.

**Figure 1:**
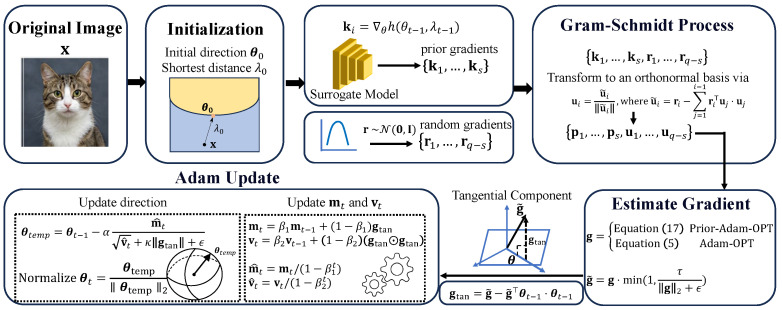
Overview of our framework. After initializing the search direction $\theta_{0}$ and boundary distance $\lambda_{0}$, an orthogonal basis is constructed using a Gram–Schmidt process combining surrogate gradients and random directions. Then, the gradient g is estimated using Equations (5) and (17) for Adam-OPT and Prior-Adam-OPT. The estimated gradient is projected onto the tangent space of the current direction $\theta_{t-1}$, and the ray direction $\theta_{t}$ is iteratively updated by using Adam-style update steps. See Algorithm 1 for details.

Unlike traditional methods that rely on simple gradient descent, Adam-OPT estimates a zeroth-order gradient by probing boundary distance variations along randomly sampled orthogonal directions. The estimated gradient is then used by Adam to update the search direction, allowing the optimizer to exploit historical gradient information and adaptive moment estimates to stabilize noisy updates. After each update, the direction is normalized to satisfy the unit-norm constraint, ensuring that the optimization remains in the directional space. Figure 1 illustrates the framework of our approach. The procedure of Adam-OPT is summarized as follows:


**Algorithm 1** Adam-OPT and Prior-Adam-OPT
1:**Input:** the original image x, the success indicator function Φ(·), number of estimation vectors *q*, numerical stability constant ϵ, step size α, first-order moment decay $\beta_{1}$, second-order moment decay $\beta_{2}$, tangent step scaling κ, number of iterations *T*, maximum gradient norm τ, surrogate model set $S=\{{\hat{\psi }}^{\left(1\right)},\ldots ,{\hat{\psi }}^{\left(s\right)}\}$ with s>0 for Prior-Adam-OPT, and S=∅ for Adam-OPT.2:**Output:** adversarial example x* that satisfies Φ(x*)=1.3:Generate 100 candidate images Ω by adding random-direction perturbations to x;4:

${\tilde{x}}_{0}\leftarrow \left\{\begin{array}{cc} \arg\min_{{\tilde{x}}_{0}\in \Omega }{∥{\tilde{x}}_{0}-x∥}_{2}\;s.t.\;\Phi \left({\tilde{x}}_{0}\right)=1, & \mathrm{untargeted}\;\mathrm{attack}\\ \mathrm{randomly}\;\mathrm{select}\;a\;\mathrm{target-class}\;\mathrm{image}\;{\tilde{x}}_{0}\;\mathrm{satisfying}\;\Phi ({\tilde{x}}_{0})=1, & \mathrm{targeted}\;\mathrm{attack} \end{array}\right.$

5:$\theta_{0}\leftarrow \frac{{\tilde{x}}_{0}-x}{∥{\tilde{x}}_{0}{-x∥}_{2}}$;6:$m_{0}\leftarrow 0$,   $v_{0}\leftarrow 0$;                                  ► Initialize first-order and second-order moments.7:**for** *t* **in** 1,…,T **do**8:    **for** ${\hat{\psi }}^{\left(i\right)}$ **in** S **do**9:        $\lambda_{t-1}\leftarrow \mathrm{BinarySearch}\left(x,\theta_{t-1},{\hat{\psi }}^{\left(i\right)},\Phi \right)$;10:     $k_{i}\leftarrow$$\nabla_{\theta }h\left(\theta_{t-1},\lambda_{t-1}\right)$ on ${\hat{\psi }}^{\left(i\right)}$ with $\lambda_{t-1}$ treated as a constant in differentiation; ► Obtain *s* transfer-based priors, where h(·,·) is defined in Equation (15).11:  **end for**12:  $r_{i}∼N\left(0,I\right)$ for i=1,…,q−s;13:  $p_{1},\ldots ,p_{s},u_{1},\ldots ,u_{q-s}\leftarrow \mathrm{Gram}–\mathrm{Schmidt}\left(k_{1},\ldots ,k_{s},r_{1},\ldots ,r_{q-s}\right)$;14:  Estimate gradient $g\leftarrow \left\{\begin{array}{cc} \mathrm{Equation}\;(17), & \mathrm{if}\;\mathrm{Prior-Adam-OPT}\\ \mathrm{Equation}\;(5), & \mathrm{if}\;\mathrm{Adam-OPT} \end{array}\right.$15:  $\tilde{g}\leftarrow g\;\cdot \;\min\left(1,\frac{\tau }{{∥g∥}_{2}+\epsilon }\right)$;                               ► Clip the gradient with the maximum norm τ.16:  $g_{\tan}\leftarrow \tilde{g}-{\tilde{g}}^{⊤}\theta_{t-1}\;\cdot \;\theta_{t-1}$                                        ► Compute the tangential gradient.17:  $m_{t}\leftarrow \beta_{1}m_{t-1}+\left(1-\beta_{1}\right)g_{\tan}$;                                          ► First-order moment.18:  $v_{t}\leftarrow \beta_{2}v_{t-1}+\left(1-\beta_{2}\right)\left(g_{\tan}⊙g_{\tan}\right)$;                                     ► Second-order moment.19:  ${\hat{m}}_{t}\leftarrow \frac{m_{t}}{1-\beta_{1}^{t}}$,   ${\hat{v}}_{t}\leftarrow \frac{v_{t}}{1-\beta_{2}^{t}}$;                                             ► Bias correction.20:  $\theta_{\mathrm{temp}}\leftarrow \theta_{t-1}-\alpha \frac{{\hat{m}}_{t}}{\sqrt{{\hat{v}}_{t}}+\kappa ∥g_{\tan}∥+\epsilon }$;                                       ► Update direction.21:  $\theta_{t}\leftarrow \frac{\theta_{\mathrm{temp}}}{∥\theta_{\mathrm{temp}}{∥}_{2}}$;                                                 ► Normalize direction.22:
**end for**
23:**return** $x*\leftarrow x+f\left(\theta_{T}\right)\frac{\theta_{T}}{∥\theta_{T}{∥}_{2}}$;

1. **Initialization:** Search for an initial adversarial direction θ and determine its boundary distance λ via binary search.
2. **Random basis construction:** Construct a set of random orthogonal directions ${\left\{u_{i}\right\}}_{i=1}^{q}$ using the Gram–Schmidt process.
3. **Zeroth-order gradient estimation:** For each perturbed direction $\theta +\sigma u_{i}$, perform binary search to estimate the corresponding boundary distance. The distance variation is used to weight each basis direction and form a zeroth-order gradient estimate.
4. **Adam-based direction update:** Update the search direction using Adam with the estimated gradient, apply gradient clipping if needed, and normalize the updated direction to maintain the unit-norm constraint.
5. **Boundary re-evaluation:** Perform binary search along the updated direction to obtain the new boundary distance, and repeat the process until the query budget or stopping criterion is reached.

#### 3.2.2. Gradient Estimation

In practical hard-label black-box attack scenarios, the gradient and confidence information of the target model are inaccessible. Therefore, Adam-OPT estimates the gradient of the objective function f(θ) using only top-1 predicted labels, where f(θ) is defined in Equation (3). Specifically, the algorithm samples *q* independent random vectors $\left\{r_{1},\ldots ,r_{q}\right\}$ from a standard Gaussian distribution and transforms them into an orthonormal basis $\left\{u_{1},\ldots ,u_{q}\right\}$ using the Gram–Schmidt process:

(4) $${\tilde{u}}_{i}=r_{i}-\sum_{j=1}^{i-1}r_{i}^{⊤}u_{j}\;\cdot \;u_{j},\;u_{i}=\frac{{\tilde{u}}_{i}}{∥{\tilde{u}}_{i}{∥}_{2}},\;i=1,\ldots ,q.$$

In Adam-OPT, gradient estimation is performed using an orthonormal basis $\left\{u_{1},\ldots ,u_{q}\right\}$, analogous to an orthogonalized variant of Sign-OPT. The key distinction is that Adam-OPT constructs the basis via Gram–Schmidt orthonormalization, whereas Sign-OPT relies on randomly sampled Gaussian vectors. Formally, the gradient is estimated as

(5) $$g:=\sum_{i=1}^{q}\mathrm{sign}\left(f\left(\theta +\sigma u_{i}\right)-f\left(\theta \right)\right)\;\cdot \;u_{i},$$

where σ is a small non-zero scalar. By leveraging the fact that the sign of a directional derivative can be estimated with a single query [4], each term $\mathrm{sign}\left(f\left(\theta +\sigma u_{i}\right)-f\left(\theta \right)\right)$ can be computed with one query as follows:

(6) $$\mathrm{sign}\left(f\left(\theta +\sigma u_{i}\right)-f\left(\theta \right)\right)=\left\{\begin{array}{cc} +1, & \mathrm{if}\;\Phi \left(x+f\left(\theta \right)\frac{\theta +\sigma u_{i}}{∥\theta +\sigma u_{i}∥}\right)=0,\\ -1, & \mathrm{otherwise}, \end{array}\right.$$

where Φ(·) is defined in Equation (2).

To improve optimization stability and suppress excessively large updates caused by noisy finite-difference estimation, we apply gradient clipping to the estimated gradient:

(7) $$\tilde{g}=g\;\cdot \;\min\left(1,\frac{\tau }{{∥g∥}_{p}+\epsilon }\right),$$

where τ denotes the maximum allowed gradient norm, *p* is the norm type used for clipping, and ϵ is a small constant for numerical stability.

#### 3.2.3. Adam Optimization of Ray Directions

After obtaining the clipped zeroth-order gradient estimate $\tilde{g}$, Adam-OPT updates the ray direction θ using an Adam-style optimizer. Since the objective f(θ) depends only on the unit $\ell_{2}$-norm search direction rather than the magnitude of θ, the optimization is performed on the unit sphere. Therefore, the radial component of the gradient, which is parallel to θ, does not contribute to changing the ray direction. To obtain a valid direction update, we first project the estimated gradient $\tilde{g}$ onto the tangent space at θ: $g_{\tan}=\tilde{g}-{\tilde{g}}^{⊤}\theta \;\cdot \;\theta$, where $g_{\tan}$ denotes the tangential gradient on the unit sphere. This projection removes the radial component and ensures that the update only changes the orientation of the ray.

**Figure 2:**
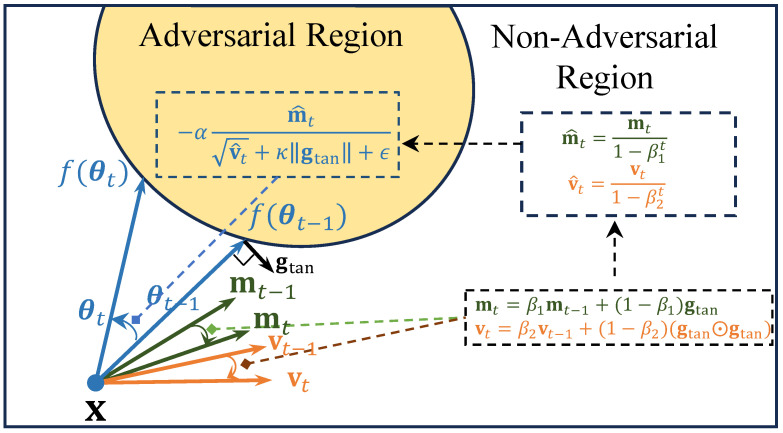
Illustration of the Adam-OPT update process. The search direction $\theta_{t}$ is iteratively updated using an Adam-style optimizer, which leverages the tangential component of the zeroth-order gradient $g_{\tan}$ to compute first- and second-order moments ($m_{t}$ and $v_{t}$) for adaptive per-parameter updates. The updated direction is then normalized to satisfy the unit-norm constraint on the hypersphere. The green, orange, and light-blue arrows denote the updates of the first-order moment $m_{t}$, the second-order moment $v_{t}$, and the ray direction $\theta_{t}$, respectively.

To further enhance optimization efficiency and stability, the algorithm incorporates the adaptive moment estimation of the Adam optimizer. The simplified two-dimensional illustration of our algorithm is shown in Figure 2.

The traditional Adam optimizer [8] internally maintains the first-order moment m (exponential moving average of gradients) and the second-order moment v (exponential moving average of squared gradients). Inspired by this, our Adam-OPT employs the tangential component $g_{\tan}$ of the estimated gradient $\tilde{g}$ to estimate the first-order and second-order moments. We first update the first-order moment estimate $m_{t}$ using an exponential weighted average of the historical gradients and the current gradient, as shown in Equation (8).

(8) $$m_{t}=\beta_{1}m_{t-1}+\left(1-\beta_{1}\right)g_{\tan},$$

where $\beta_{1}$ is the first-order moment decay rate. The first-order moment estimate in Equation (8) smooths the optimization trajectory by aggregating historical gradients with the current gradient through exponential moving averaging. As a result, Adam-OPT is less sensitive to occasional noisy or unstable gradient estimates, while consistently aligned gradients across successive iterations are progressively reinforced, leading to a more stable and reliable optimization direction. Then, we apply Equation (9) to update the second-order moment $v_{t}$:

(9) $$v_{t}=\beta_{2}v_{t-1}+\left(1-\beta_{2}\right)\left(g_{\tan}⊙g_{\tan}\right),$$

where $\beta_{2}$ is the second-order moment decay rate, and ⊙ denotes the element-wise product. The second-order moment estimate in Equation (9) adaptively tracks the magnitude of the tangential gradients via exponential averaging of their squared values, allowing Adam-OPT to dynamically adjust the effective update scale for each dimension. Specifically, dimensions with consistently large gradients are assigned smaller effective update steps, while dimensions with relatively small gradients receive comparatively larger adaptive updates. This adaptive scaling mechanism improves optimization stability under noisy zeroth-order gradient estimation. The third step is the bias correction, which uses Equations (10) and (11) to correct the issue of overly small estimates in previous steps:

(10) $$\begin{array}{cc} {\hat{m}}_{t} & =\frac{m_{t}}{1-\beta_{1}^{t}}, \end{array}$$

(11) $$\begin{array}{cc} {\hat{v}}_{t} & =\frac{v_{t}}{1-\beta_{2}^{t}}. \end{array}$$

Bias correction compensates for the initialization bias of the moment estimates in early iterations, thereby providing more accurate adaptive update directions and improving optimization stability. Finally, we use Equation (12) to update the ray direction of Adam-OPT:

(12) $$\theta_{\mathrm{temp}}=\theta_{t-1}-\alpha \frac{{\hat{m}}_{t}}{\sqrt{{\hat{v}}_{t}}+\kappa ∥g_{\tan}∥+\epsilon },$$

where α is the step size, ϵ is a numerical stability constant, and $\kappa ∥g_{\tan}∥$ regulates the update step size and prevents overly large updates on the unit sphere, which may lead to overshooting the local optimum during direction optimization. Since the updated vector $\theta_{\mathrm{temp}}$ generally deviates from the unit sphere, we further enforce the constraint $∥\theta_{t}{∥}_{2}=1$ through normalization:

(13) $$\theta_{t}=\frac{\theta_{\mathrm{temp}}}{∥\theta_{\mathrm{temp}}{∥}_{2}}.$$

In all experiments, we set the hyperparameters as α=0.4, $\beta_{1}=0.9$, $\beta_{2}=0.999$, $\epsilon =10^{-8}$, and κ=0.1. The above procedure constitutes the core optimization loop for ray-direction search on the unit sphere. Specifically, the tangential update removes the radial component of the gradient to ensure that the optimization focuses only on direction changes, while the normalization step is applied to project the updated $\theta_{\mathrm{temp}}$ back onto the unit sphere. Built upon this geometry-aware optimization framework, Adam-OPT further leverages the adaptive moment estimation mechanism of Adam to achieve more stable and reliable zeroth-order optimization.

#### 3.2.4. Boundary Distance Estimation via Two-Stage Search

For a given normalized ray direction θ, Adam-OPT evaluates the objective function value f(θ) by estimating the minimum perturbation magnitude required to reach the adversarial region along this direction. Specifically, f(θ) is defined as in Equation (3), i.e., $f\left(\theta \right)=\inf\left\{\lambda >0:\Phi \left(x+\lambda \frac{\theta }{∥\theta ∥}\right)=1\right\}$, where Φ(·) is the attack success indicator function defined in Equation (2).

This boundary distance estimation is required whenever Adam-OPT evaluates a ray direction. During initialization, after a valid adversarial direction is found, the algorithm refines its boundary distance to obtain the initial objective value $f(\theta_{0})$. During iterative optimization, after the direction update to $\theta_{t+1}$, the same procedure is used to estimate the new objective value $f(\theta_{t+1})$. Since confidence scores and gradients of the target model are unavailable, Adam-OPT can only determine whether a queried point x+λθ lies inside or outside the adversarial region according to its top-1 predicted label, and thus estimates the boundary distance through a two-stage search procedure.

The search procedure consists of two stages. The first stage constructs an interval $\left[\lambda_{\mathrm{lo}},\lambda_{\mathrm{hi}}\right]$ that contains the decision boundary. Given an initial distance estimate λ, Adam-OPT first checks the prediction of x+λθ. For untargeted attacks, if this point is still classified as the original label, then λ is regarded as a lower bound, and the algorithm progressively enlarges the upper bound until misclassification occurs. Otherwise, if x+λθ is already adversarial, then λ is regarded as an upper bound, and the algorithm progressively decreases the lower bound until the prediction returns to the original label. For targeted attacks, the same bracketing strategy is applied using the target-label condition: the upper bound is enlarged until the target class is reached, or the lower bound is reduced while the perturbed image remains classified as the target class.

After a valid interval is obtained, Adam-OPT performs binary search within $\left[\lambda_{\mathrm{lo}},\lambda_{\mathrm{hi}}\right]$ to refine the boundary distance estimate. At each iteration, the midpoint

(14) $$\lambda_{\mathrm{mid}}=\frac{\lambda_{\mathrm{lo}}+\lambda_{\mathrm{hi}}}{2}$$

is evaluated by querying the prediction of $x+\lambda_{\mathrm{mid}}\theta$. For untargeted attacks, if the midpoint already causes misclassification, it becomes the new upper bound; otherwise, it becomes the new lower bound. For targeted attacks, if the midpoint is classified as the target label, it becomes the new upper bound; otherwise, it becomes the new lower bound. This process is repeated until the interval length is smaller than a prescribed tolerance, and the final upper bound is used as the estimated value of f(θ).

In practice, different tolerances can be used for different purposes. A relatively coarse tolerance is sufficient when the boundary search is used inside zeroth-order gradient estimation, while a finer tolerance can be adopted when evaluating the updated ray direction. To avoid degenerate search behavior, Adam-OPT further includes numerical safeguards, such as stopping the search when the interval can no longer be refined due to floating-point precision or when the number of search iterations exceeds a predefined limit. These mechanisms provide a stable and query-efficient estimate of the ray-based objective function value under hard-label feedback.

### 3.3. Prior-Guided Adam Optimization for Ray Direction Search

Although Adam-OPT benefits from momentum and adaptive optimization, the gradient estimation still relies on purely random exploration directions, without exploiting more informative transfer-based priors from surrogate models. Consequently, its performance is suboptimal even under a budget of 2000 queries. To address this, we introduce a transfer-based prior into Adam-OPT, resulting in the Prior-Adam-OPT algorithm.

Unlike Adam-OPT, Prior-Adam-OPT leverages surrogate models to provide more informative transfer-based priors, thereby enhancing gradient estimation. We assume that the adversary has access to several white-box surrogate models, from which gradients can be computed via backpropagation. However, a key challenge remains: even for white-box models, the gradient of the objective function f(θ), defined in Equation (3), cannot be directly obtained because f(θ) is evaluated via the non-differentiable binary search procedure described in Section 3.2.4. To address this issue, we adopt a differentiable surrogate function h(θ,λ), following Ma et al. [5], defined as

(15) $$\begin{array}{c} h\left(\theta ,\lambda \right):=\left\{\begin{array}{cc} {\hat{\psi }}_{y}-\max_{j\neq y}{\hat{\psi }}_{j}, & \mathrm{for}\;\mathrm{untargeted}\;\mathrm{attacks},\\ \max_{j\neq {\hat{y}}_{\mathrm{adv}}}{\hat{\psi }}_{j}-{\hat{\psi }}_{{\hat{y}}_{\mathrm{adv}}}, & \mathrm{for}\;\mathrm{targeted}\;\mathrm{attacks}, \end{array}\right. \end{array}$$

where ${\hat{\psi }}_{i}:=\hat{\psi }{\left(x+\lambda \;\cdot \;\frac{\theta }{∥\theta ∥}\right)}_{i}$ denotes the *i*-th output element of the surrogate model $\hat{\psi }$, x is the original image, *y* denotes the true label, and ${\hat{y}}_{\mathrm{adv}}$ denotes the target class determined for the surrogate model in targeted attacks. The surrogate function h(θ,λ) effectively corresponds to the negative Carlini–Wagner (C&W) loss evaluated at the surrogate model. Its key property is that, for any non-zero vector $\theta_{0}\in R^{d}$ with finite objective $f(\theta_{0})<+\infty$, the gradient of the true objective function f(θ) is proportional to the gradient of the surrogate:

(16) $$\nabla f\left(\theta_{0}\right)=c\;\cdot \;\nabla_{\theta }h\left(\theta_{0},\lambda_{0}\right),$$

where $\lambda_{0}=f\left(\theta_{0}\right)$ is treated as a constant during differentiation, and *c* is a non-zero scalar. The specific value $\lambda_{0}$ is obtained via binary search along the ray defined by $\theta_{0}$, such that $h(\theta_{0},\lambda_{0})$ measures the surrogate loss at the decision boundary. The proof of Equation (16) is provided in Ma et al. [5].

Suppose we have *s* surrogate models, from which we obtain *s* prior directions via $\nabla_{\theta }h\left(\theta_{0},\lambda_{0}\right)$. We denote these prior directions as $k_{1},\ldots ,k_{s}$. In addition, we randomly sample q−s Gaussian vectors $r_{1},\ldots ,r_{q-s}$ to complement the prior space.

We then apply the Gram–Schmidt process with Equation (4) to orthogonalize and normalize the combined set of vectors $k_{1},\ldots ,k_{s},r_{1},\ldots ,r_{q-s}$, resulting in an orthonormal basis $p_{1},\ldots ,p_{s},u_{1},\ldots ,u_{q-s}$. Here, $p_{1},\ldots ,p_{s}$ correspond to the prior directions $k_{1},\ldots ,k_{s}$, while $u_{1},\ldots ,u_{q-s}$ correspond to the random directions $r_{1},\ldots ,r_{q-s}$. Our objective is then to estimate the projection of the gradient onto the subspace *S* spanned by this orthonormal basis using as few queries as possible. In Prior-Adam-OPT, we adopt a gradient estimation procedure of Prior-OPT [5]. Specifically, the gradient is approximated as

(17) $$g:=\sum_{i=1}^{s}\frac{f\left(\theta +\sigma p_{i}\right)-f\left(\theta \right)}{\sigma }\;\cdot \;p_{i}+\frac{f\left(\theta +\sigma \bar{g_{⊥}}\right)-f\left(\theta \right)}{\sigma }\;\cdot \;\bar{g_{⊥}},$$

where $\bar{g_{⊥}}$ denotes the $\ell_{2}$-normalized vector of $g_{⊥}$, and $g_{⊥}$ is computed as

(18) $$g_{⊥}:=\sum_{i=1}^{q-s}\mathrm{sign}\left(f\left(\theta +\sigma u_{i}\right)-f\left(\theta \right)\right)\;\cdot \;u_{i},$$

where each term $\mathrm{sign}\left(f\left(\theta +\sigma u_{i}\right)-f\left(\theta \right)\right)$ can be estimated with one query by using Equation (6).

Once the estimated gradient g is obtained, we first apply the gradient clipping technique in Equation (7) to obtain the clipped gradient $\tilde{g}$. We then project $\tilde{g}$ onto the tangent space at θ: $g_{\tan}=\tilde{g}-\left({\tilde{g}}^{⊤}\theta \right)\theta$, where $g_{\tan}$ denotes the tangential gradient on the unit sphere.

Subsequent updates follow the same procedure as in Adam-OPT, i.e., we perform Adam-based updates according to Equations (8)–(13). The complete workflow of Adam-OPT and Prior-Adam-OPT is summarized in Algorithm 1.

## 4. Experiment

### 4.1. Experimental Setting

#### 4.1.1. Implementation Details and Datasets

In this paper, all experiments are conducted using the PyTorch 1.7.1 framework with Python 3.7.6 on NVIDIA V100 GPUs (Nvidia Corporation, Santa Clara, CA, USA). The source code and implementation details of our approach are publicly available at https://github.com/machanic/hard_label_attacks (accessed on 1 May 2026). We conduct experiments on two standard datasets, namely CIFAR-10 [9] and ImageNet [10]. The image dimensions are 32×32×3 for CIFAR-10, and either 299×299×3 or 224×224×3 for ImageNet, depending on the target model architecture. The hyperparameters used in the experiments are shown in Table 1.

#### 4.1.2. Compared Methods

To validate the effectiveness of our proposed approach, we select several state-of-the-art baseline methods for comparison, including HopSkipJumpAttack (HSJA) [16], Tangent Attack (TA) [15], Sign-OPT [4], Evolutionary [11], GeoDA [12], SurFree [13], AHA [19], Prior-Sign-OPT [5], and Prior-OPT [5]. For methods leveraging surrogate models, the specific surrogate model used is indicated via a subscript; for example, Prior-OPT_ConViT_ denotes that ConViT [21] serves as the surrogate model.

#### 4.1.3. Evaluation Metric

We randomly select 1000 images from each dataset to evaluate our approach. To ensure a fair comparison, for a given query budget *Q*, the distortion of each image x is defined as the minimal $\ell_{2}$ perturbation obtained from all queries up to *Q*, i.e., $D_{Q}\left(x\right):=\min_{q\leq Q}{∥x_{\mathrm{adv}}\left(q\right)-x∥}_{2}$, where $x_{\mathrm{adv}}\left(q\right)$ denotes the adversarial example obtained after *q* queries. The mean distortion is then computed over all selected images as $\frac{1}{\left|X\right|}\sum_{x\in X}D_{Q}\left(x\right)$, where *X* denotes the set of selected images. The evaluation is performed under different query budgets ranging from 400 to 2000. Additionally, the attack success rate (ASR) under a given query budget *Q* is defined as the percentage of images for which the minimal $\ell_{2}$ perturbation obtained within *Q* queries is less than or equal to the threshold $\delta =\sqrt{0.0015\times d}$, where *d* is the image dimension.

### 4.2. Comparison with State-of-the-Art Attacks

On the CIFAR-10 dataset, we use ResNet-110 as the surrogate model for Prior-Sign-OPT, Prior-OPT, and Prior-Adam-OPT. On the ImageNet dataset, for surrogate-model-based methods (Prior-Sign-OPT, Prior-OPT, and Prior-Adam-OPT), we employ ResNet-50 as the surrogate for the ResNet-101 target model, and ConViT [21] as the surrogate for the Swin Transformer [22] target model.

#### 4.2.1. Results of Attacks Against Undefended Models

Table 2 reports the mean $\ell_{2}$ distortions of untargeted attacks on the ImageNet dataset across varying query budgets. For both the ResNet-101 and Swin Transformer target models, Prior-Adam-OPT consistently achieves the smallest distortions when the query budget exceeds 800, outperforming all baseline methods overall. The standard Adam optimizer converges quickly because it adaptively scales the learning rate for each parameter using estimates of both the first-order and second-order moments of the gradients. This per-parameter adaptation allows larger steps in directions of low variance and smaller steps in directions of high variance, stabilizing updates and accelerating convergence.

The proposed Prior-Adam-OPT leverages these advantages while further incorporating transfer-based priors from surrogate models, resulting in significantly lower $\ell_{2}$ distortions than Prior-OPT under the same query budgets. This demonstrates that combining a more sophisticated optimization algorithm with transfer-based prior information can substantially improve attack efficiency. Notably, for both ResNet-101 and Swin Transformer target models, Prior-Adam-OPT achieves consistently smaller distortions for most query budgets compared to classical hard-label attacks and surrogate-model-based baselines. These results highlight the effectiveness of our approach in generating high-quality adversarial examples with minimal perturbation, emphasizing that optimization strategy and prior knowledge jointly contribute to superior attack performance.

Table 3 reports the mean $\ell_{2}$ distortions of untargeted attacks on CIFAR-10, with WRN-40 as the target model and ResNet-110 as the surrogate model. Adam-OPT outperforms Sign-OPT when the number of queries exceeds 1600; neither method uses surrogate models, highlighting the advantage of employing a more sophisticated optimization algorithm. Prior-Adam-OPT, which combines Adam’s adaptive per-parameter updates with transfer-based priors, generally achieves smaller distortions than Adam-OPT across most query budgets and surpasses Prior-OPT for query budgets of 1600 and above. This demonstrates that integrating advanced optimization with surrogate model knowledge enables the generation of high-quality adversarial examples with reduced perturbations.

To evaluate the scalability of our proposed approach, we perform untargeted attacks on the CLIP model with the ViT-L/14 backbone. Table 4 reports the mean $\ell_{2}$ distortions and attack success rates under varying query budgets. Prior-Adam-OPT generally achieves lower distortions than Adam-OPT across most query budgets and exhibits the lowest distortions at higher query budgets. Notably, Prior-Adam-OPT achieves the highest attack success rates of 65.2% and 69.3% under query budgets of 1600 and 2000, respectively, demonstrating its superior attack effectiveness. These results highlight that incorporating the surrogate model as a prior substantially improves attack performance, even though the surrogate model is pretrained on ImageNet and differs fundamentally from CLIP’s contrastive language-image pretraining. The ability of Prior-Adam-OPT to leverage knowledge from surrogate models of different architectures underscores the benefit of combining adaptive optimization with transfer-based priors, enabling effective attacks against zero-shot classifiers such as CLIP.

#### 4.2.2. Results of Attacks Against Defense Models

**Figure 3:**
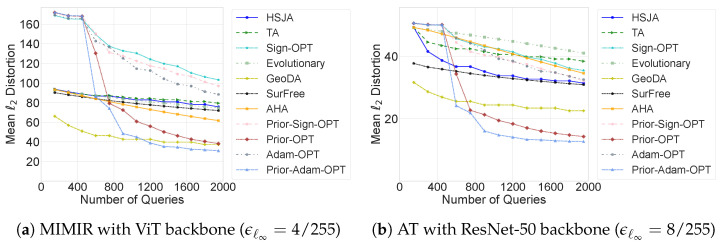
Mean distortions of $\ell_{2}$-norm untargeted attacks against defense models. The surrogate model of Prior-Adam-OPT is an adversarially trained ResNet-50 ($\epsilon_{\ell_{\infty }}=4/255$).

We evaluate untargeted attacks against two types of defense models: adversarial training (AT) [23] and MIMIR [24], where MIMIR achieves state-of-the-art robustness on RobustBench [25]. Figure 3 shows the mean $\ell_{2}$ distortions of various attack methods on ImageNet across different query budgets, with Prior-Sign-OPT, Prior-OPT, and Prior-Adam-OPT using an adversarially trained ResNet-50 as the surrogate model. Prior-Adam-OPT generally outperforms Adam-OPT across most query budgets. Its advantage becomes more pronounced at higher query budgets. In particular, under query budgets exceeding 1200 against MIMIR and 800 against AT, Prior-Adam-OPT achieves the lowest distortion among all methods. This trend indicates that the full potential of Prior-Adam-OPT emerges in the later stages of the attack process, where the combination of per-parameter adaptive updates and transfer-based priors allows for more effective and efficient attacks. Consequently, Prior-Adam-OPT achieves superior performance against both AT and MIMIR, particularly when sufficient queries are available to fully leverage its optimization and transfer-based priors.

### 4.3. Ablation Studies of Prior-Adam-OPT

**Figure 4:**
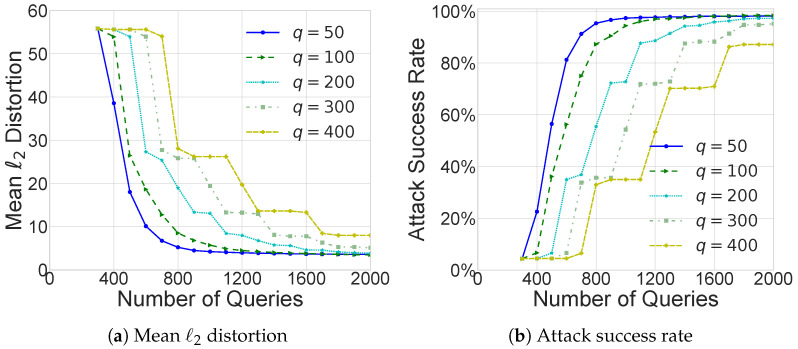
Effect of the number of vectors *q* used for gradient estimation. Experiments are conducted using Prior-Adam-OPT for untargeted attacks on ResNet-101, with ResNet-50 as the surrogate model.

**Figure 5:**
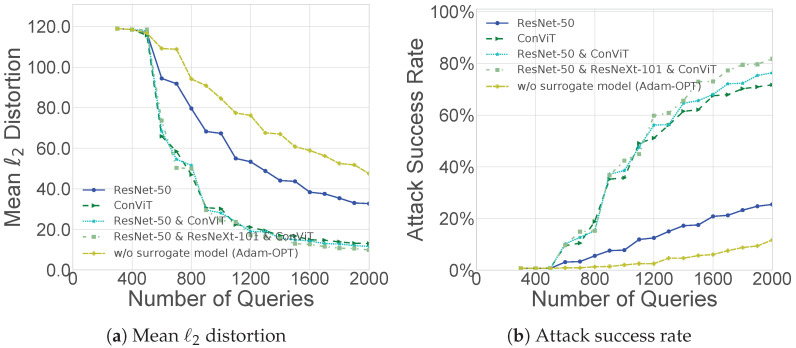
Impact of surrogate model choice. We conduct untargeted attacks on Swin Transformer using Prior-Adam-OPT, with Adam-OPT used for results without a surrogate model, on ImageNet.

Figure 4 and Figure 5 present ablation studies of Prior-Adam-OPT, examining two factors: the number of vectors used for gradient estimation and the choice of surrogate models.

The number of vectors used for gradient estimation, denoted as *q*, balances two competing objectives. If *q* is too large, gradient estimates become more accurate, but the query budget is quickly depleted; if *q* is too small, the estimated gradients are unreliable, making the attack more sensitive to discrepancies between the surrogate and target models. To identify an appropriate trade-off, we conducted ablation experiments with varying values of *q*, as shown in Figure 4. Based on these results, we set q=200 in our main experiments, which provides a robust compromise between gradient precision and query efficiency.

Similarly, the number and quality of surrogate models directly affect the effectiveness of the prior directions $p_{1},\ldots ,p_{s}$. Surrogate models with higher architectural similarity to the target model provide more informative priors, improving search efficiency, whereas dissimilar or low-quality surrogates may mislead the search. Figure 5 shows that surrogate model architectures more similar to the target model’s architecture yield better attack performance, and using multiple surrogate models further improves performance compared to using a single surrogate or none at all. Even when a surrogate model (ResNet-50) differs substantially from a Swin Transformer target model, its contribution is smaller than that of ConViT, which is more similar, yet it still enhances performance compared to the baseline without any surrogate model. These results highlight that both the number and quality of surrogate models are crucial for guiding Prior-Adam-OPT to perform efficient attacks.

## 5. Conclusions

In this work, we proposed two query-efficient hard-label black-box attack methods: Adam-OPT and its prior-guided variant Prior-Adam-OPT. Adam-OPT introduces an Adam-based adaptive optimization strategy to the ray-search framework, leveraging historical gradient information and per-parameter moment estimates to stabilize zeroth-order gradient updates and accelerate convergence. Building upon this, Prior-Adam-OPT incorporates transfer-based priors from surrogate models into the gradient estimation process, constructing an orthogonal basis that combines prior and random directions to further improve gradient accuracy and query efficiency. Extensive experiments on CIFAR-10, ImageNet, and zero-shot CLIP classifiers demonstrate that both methods achieve competitive performance, with Prior-Adam-OPT consistently outperforming Adam-OPT and existing baselines in terms of $\ell_{2}$ distortion and attack success rate, particularly under larger query budgets. Ablation studies highlight the importance of selecting an appropriate number of vectors for estimating gradients and high-quality surrogate models. Overall, our results indicate that combining adaptive optimization with transfer-based priors enables more precise and efficient generation of adversarial examples, providing a robust framework for hard-label black-box attacks in high-dimensional and real-world scenarios.

## Figures and Tables

**Table 1:** Hyperparameter Settings.

Hyperparameter	Description	Default Value
α	Step size	0.4
*q*	Number of vectors for gradient estimation	200
tol (θ)	Binary search stopping threshold for computing f(θ) along the updated search direction θ	$1\times 10^{-4}$
tol (g)	Binary search stopping threshold used in gradient estimation (Equation (17))	$1\times 10^{-3}$
$Q_{\max}$	Maximum number of queries	2000
δ	Attack success threshold	$\sqrt{0.0015\times d}$
$\beta_{1}$	First-order moment decay	0.9
$\beta_{2}$	Second-order moment decay	0.999
κ	Tangent step scaling used in Equation (12)	0.1

**Table 2:** Mean $\ell_{2}$ distortions across query budgets on the ImageNet dataset. ResNet-50 and ConViT are used as the surrogate models for attacks against ResNet-101 and Swin Transformer, respectively.

Model	Method	$\ell_{2}$-Norm Attacks				
		@0.4K	@0.8K	@1.2K	@1.6K	@2.0K
ResNet-101	HSJA [16]	46.014	32.453	27.552	20.904	18.388
	TA [15]	43.970	31.725	27.355	20.919	18.491
	Sign-OPT [4]	55.486	41.447	32.677	25.269	21.235
	Evolutionary [11]	45.459	35.757	29.421	24.905	21.507
	GeoDA [12]	**27.287**	21.984	17.936	14.871	12.526
	SurFree [13]	33.594	27.029	22.984	20.183	18.100
	AHA [19]	49.025	38.763	31.987	27.021	23.180
	Prior-Sign-OPT_ResNet-50_ [5]	55.825	38.644	30.740	23.597	18.733
	Prior-OPT_ResNet-50_ [5]	55.440	22.623	14.812	9.885	7.100
	Adam-OPT	55.643	43.436	33.441	24.779	19.314
	Prior-Adam-OPT_ResNet-50_	55.643	**19.030**	**7.953**	**4.739**	**3.873**
Swin Transformer	HSJA [16]	94.603	74.643	65.951	51.505	45.823
	TA [15]	91.369	73.236	65.470	52.060	46.728
	Sign-OPT [4]	118.726	94.236	77.015	63.140	53.399
	Evolutionary [11]	89.621	74.903	64.182	55.829	49.243
	GeoDA [12]	65.282	56.694	48.943	42.221	36.722
	SurFree [13]	**64.720**	51.767	43.783	38.224	34.281
	AHA [19]	89.964	72.965	61.326	53.018	46.756
	Prior-Sign-OPT_ConViT_ [5]	117.684	88.613	72.624	58.167	46.623
	Prior-OPT_ConViT_ [5]	118.599	52.705	35.009	24.731	19.482
	Adam-OPT	118.493	94.161	76.193	58.907	47.510
	Prior-Adam-OPT_ConViT_	118.493	**46.994**	**21.088**	**14.909**	**13.036**

**Table 3:** Mean $\ell_{2}$ distortions across query budgets on the CIFAR-10 dataset. ResNet-110 is used as the surrogate model for Prior-Sign-OPT, Prior-OPT, and Prior-Adam-OPT.

Model	Method	$\ell_{2}$-Norm Attacks				
		@0.4K	@0.8K	@1.2K	@1.6K	@2.0K
WRN-40-10 (with dropout)	HSJA [16]	**1.830**	**0.973**	**0.779**	0.564	0.499
	TA [15]	1.868	0.981	**0.779**	**0.560**	0.495
	Sign-OPT [4]	4.667	3.495	2.949	2.337	1.827
	Evolutionary [11]	2.683	1.801	1.343	1.063	0.879
	GeoDA [12]	2.707	1.967	1.604	1.426	1.270
	SurFree [13]	2.141	1.557	1.247	1.061	0.938
	AHA [19]	3.696	2.848	2.434	2.212	2.088
	Prior-Sign-OPT_ResNet-110_ [5]	4.773	2.921	1.805	1.119	0.803
	Prior-OPT_ResNet-110_ [5]	4.199	2.322	1.473	0.837	0.572
	Adam-OPT	9.865	8.240	2.960	0.823	0.615
	Prior-Adam-OPT_ResNet-110_	9.865	7.923	2.900	0.836	**0.447**

**Table 4:** Experimental results of attacks against CLIP with the ViT-L/14 backbone. ConViT is used as the surrogate model for Prior-Sign-OPT, Prior-OPT, and Prior-Adam-OPT.

Method	Mean $\ell_{2}$ Distortions					Attack Success Rate ^1^				
	@0.4K	@0.8K	@1.2K	@1.6K	@2.0K	@0.4K	@0.8K	@1.2K	@1.6K	@2.0K
HSJA [16]	63.055	61.378	60.652	59.586	58.948	7.1%	8.1%	8.4%	9.2%	9.8%
TA [15]	61.201	59.446	58.651	57.751	56.892	7.7%	9.1%	9.4%	10.2%	10.6%
Sign-OPT [4]	65.846	60.060	55.929	52.019	49.435	14.9%	16.0%	16.7%	17.6%	17.9%
Evolutionary [11]	50.541	37.412	29.049	23.263	19.206	9.9%	17.9%	28.3%	39.5%	48.1%
GeoDA [12]	46.546	44.588	43.152	41.608	40.114	11.8%	13.2%	14.6%	16.5%	18.6%
SurFree [13]	**30.594**	**22.889**	**18.362**	15.329	**13.196**	**31.0%**	**44.1%**	**55.4%**	62.2%	68.9%
AHA [19]	54.326	41.682	33.057	27.305	22.925	7.3%	12.2%	20.9%	29.7%	37.5%
Prior-Sign-OPT_ConViT_ [5]	65.461	57.747	51.486	47.111	43.327	14.4%	15.8%	17.3%	19.1%	20.4%
Prior-OPT_ConViT_ [5]	65.846	38.044	28.580	23.930	21.267	14.8%	28.7%	39.5%	46.1%	51.6%
Adam-OPT	66.611	52.449	39.925	28.379	21.488	14.4%	20.5%	27.8%	37.2%	49.0%
Prior-Adam-OPT_ConViT_	66.529	33.620	20.678	**15.278**	13.326	14.4%	36.4%	52.4%	**65.2%**	**69.3%**

^1^ The distortion threshold for the attack success rate is 15.026, which is calculated as $\sqrt{0.0015\times 224\times 224\times 3}$.

## Data Availability

The source code and implementation details supporting the findings of this study are publicly available at https://github.com/machanic/hard_label_attacks (accessed on 1 May 2026). Other experimental data are available from the corresponding author upon reasonable request.
